# Relationship between quantum speed limit time and memory time in a photonic-band-gap environment

**DOI:** 10.1038/srep39110

**Published:** 2016-12-23

**Authors:** J. Wang, Y. N. Wu, M. L. Mo, H. Z. Zhang

**Affiliations:** 1 School of physics and technology, University of Jinan, Jinan, 250022, China; 2 College of physics, Jilin University, Changchun, 130023, China

## Abstract

Non-Markovian effect is found to be able to decrease the quantum speed limit (QSL) time, and hence to enhance the intrinsic speed of quantum evolution. Although a reservoir with larger degree of non-Markovianity may seem like it should cause smaller QSL times, this seemingly intuitive thinking may not always be true. We illustrate this by investigating the QSL time of a qubit that is coupled to a two-band photonic-band-gap (PBG) environment. We show how the QSL time is influenced by the coherent property of the reservoir and the band-gap width. In particular, we find that the decrease of the QSL time is not attributed to the increasing non-Markovianity, while the memory time of the environment can be seen as an essential reflection to the QSL time. So, the QSL time provides a further insight and sharper identification of memory time in a PBG environment. We also discuss a feasible experimental realization of our prediction.

In quantum information and communication theory, a central objective is to know how fast can a quantum system evolve so as to develop the ultra-speed communication channel and quantum computer. The intrinsic minimal evolution time between two states, known as quantum speed limit (QSL) time^1^, is a key method in characterizing the maximal rate of quantum evolution. For closed quantum systems, the QSL time is determined by the Mandelstam-Tamm (MT) type bound and the Margolus-Levitin (ML) type bound^2^,^3^.

Over the past few years, a considerable amount of work has been devoted to the extensions of the QSL time for open quantum systems^4^,^5^,^6^,^7^, since the interaction between quantum systems and environments can not be ignored. Several new QSL time bounds for open quantum systems have been formulated^8^. The analysis of the environmental effects on the QSL time has been recently applied to a number of systems such as spin-boson models^9^,^10^, atoms in photonic crystals^11^, and spin chains^12^.

Interestingly, it is found that the non-Markovian evolution induced by the memory effect of environment can accelerate the quantum evolution^13^,^14^,^15^,^16^. A good example of this is the situation where an atom is coupled to a leaky single mode cavity^13^. For this model, it has been discovered that increasing non-Markovianity will decrease the QSL time, and therefore lead to a faster speed of the intrinsic evolution. Moreover, a monotonic relationship between the non-Markovianity and the QSL time is presented in different settings^17^,^18^. One question naturally arise: whether can the degree of non-Markovianity directly reflect the length of QSL time in a memory environment.

The purpose of this paper is to examine the relation between non-Markovianity and QSL time in a environment with memory effects. To do so, we will study the QSL time of a single two-level atom that is immersed in a coherent photonic crystal consisting of an upper band, a lower band and a band gap^19^,^20^,^21^. The environmental coherence means that the lower-band and upper-band reservoirs are correlated and can become coherent due to the definite phase difference between the fields from the double-band reservoir. This interaction between atomic system and the PBG reservoir has been widely used to form population trapping^22^, spontaneous emission suppression^23^,^24^,^25^ and quantum entanglement preservation^26^,^27^,^28^,^29^,^30^,^31^,^32^,^33^,^34^.

In this setting, we investigate how a decrease of QSL time can be acquired by manipulating the environmental coherent property and the band-gap width. It is shown that the environmental coherence and the width of the band gap, all serve to reduce the QSL time in some cases. For the mechanism of intrinsic quantum speedup, some nontrivial and unexpected results are found. The non-Markovianity can not directly affect the QSL time. In other words, a larger value of the non-Markovianity does not necessarily result in a shorter QSL time. So in order to clear the physical reason, we further focus on the relation between the QSL time and the memory time of the environment. The memory time of the PBG environment plays a decisive role in the memory effect, and can be characterized by the time taken by some information to travel from the system to the environment and back^35^. We find that the QSL time reduction is attributed to the decreasing memory time of environment, which indicates the increase of the return velocity of information. It is not the non-Markovianity, i.e., the total amount of backflow information, but the backflow rate of information can be seen as an essential reflection to the QSL time. Our results suggest that the QSL time can witness the memory time of PBG environments.

In the following, we will first present a model in which a qubit is coupled to a coherent double-band PBG environment. Secondly, we will consider the environmental effects on the QSL time. The relation between the QSL time and the memory time of the PBG environment will also be explored.

## Results

### The system-environment model

The global system we consider comprises a two-band photonic crystal containing a single two-level atom placed at location **r**_0_. The corresponding Hamiltonian is (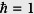)


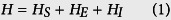


with the system Hamiltonian


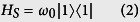


describing a two-level system with the excited state 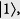 ground state 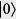 and transition frequency *ω*_0_. The Hamiltonian of the double-band photonic crystal environment,


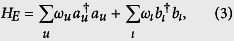


represents a environment of harmonic oscillators with field operator 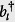 (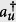) for the lower (upper) band PBG reservoir. The interaction Hamiltonian reads


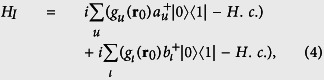


where 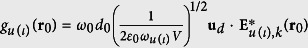 is the position-dependent coupling constant of the two-level atom with the upper (lower) band PBG reservoir modes with frequency *ω*_*u(ι*)_. Here, *d*_0_ refers to the magnitude of the dipolar moment and its direction is represented by **u**_*d*_. 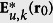 and 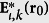 represent the eigenmode fields from the two-band PBG reservoir. It has been proved that the eigenmode fields depend on the atomic embedded position and can interfere with each other^36^. Thus, we can write the fields 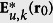 and 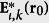 as^36^


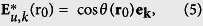



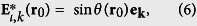


where **e**_**k**_ is the unit vector of the electric field. The parameter *θ*(**r**_0_) represents the angle seen by the two-level atom placed at **r**_0_. The model thus describes the coupling of a qubit to a PBG reservoir with coherent property, which results from the *π*/2 phase difference between fields 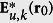 and 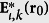.

We assume that the atom is initially excited, and the two reservoir modes are in the vacuum state 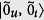. The state of the total system at time *t* takes the form


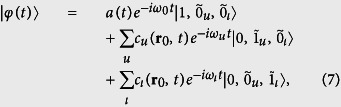


where 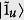 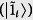 is the radiation state containing one excitation only in the *u*th (*ι*th) mode.

The probability amplitudes of the system are governed by the Schrödinger equation, from which we can obtain


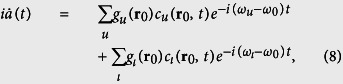



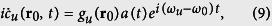



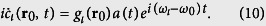


Eliminating *c*_*u*_(**r**_0_, *t*) and *c*_*ι*_(**r**_0_,*t*) in Eq. (8), one finds


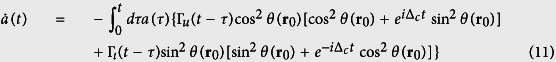


where 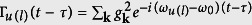 represents the memory kernels from the upper (lower)-band reservoir. Here, we have applied *g*_*u*_(**r**_**0**_) ≅ *g*_**k**_ cos *θ*(**r**_0_) and *g*_*l*_(**r**_**0**_) ≅ *g*_**k**_ sin *θ*(**r**_0_) with constant 
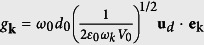
, *c*_*u*_(**r**_0_, *t*) = *c*_**k**_(*t*) cos *θ*(**r**_0_), *c*_*ι*_(**r**_0_, *t*) = *c*_**k**_(*t*) sin *θ*(**r**_0_), and the band-gap width 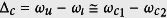 with the upper (lower) band edge frequency 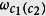.

The Laplace transform of *a(t*) is


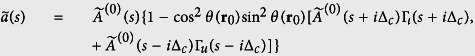


with 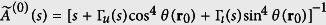. Γ_*u(ι*)_(*s*) represents the Laplace transform of Γ_*u(ι*)_(*t* − *τ*) and its calculation is shown in the method section. For the analytical result of *a(t*) we refer the reader to the Supplementary Material (see the Eq. (S7)).

In the above discussion, we only considered a coherence case, where the PBG reservoir is coherent resulting from the *π*/2 phase difference between the lower-band and upper-band fields of PBG (see Equations (5) and (6)). Now, we study a bit of more general case, where the two-band PBG reservoir is incoherent, i.e., the eigenmode fields from the lower-band and upper-band reservoirs are incoherent waves and independent of the atomic embedded position [*θ(r*_0_)]. Thus, the coupling strengths of the atom with the lower-band and upper-band reservoir modes are the same and equal to the constant 
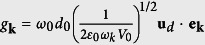
. In the non-coherence case, the Laplace transform of the probability amplitude in the state 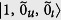 can be given by 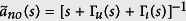. The calculation process of 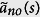 is shown in detail in the Re^37^. The analytical result of the amplitude *a*_*no*_(*t*) for no coherence case is shown in the Supplementary Material.

### Quantum speed limit time

The aim of this section is to investigate the QSL time problem of a two-level system interacting with a PBG environment. The QSL time is defined as the intrinsic minimal time a system evolving between two states. A unified expression for the QSL time in open systems, widely used to evaluate the speed of quantum evolution, can be written as^13^


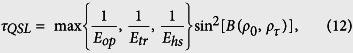


where 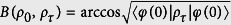 is the Bures angle between the target state *ρ*_*τ*_ and the initial pure state 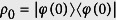. 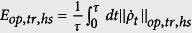, with 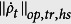 denoting the operator norm, trace norm and Hilbert-Schmidt norm of 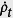, respectively. Using this QSL time bound, we can evaluate the intrinsic speed of the dynamical evolution by a given driving time *τ*. When the QSL time *τ*_*QSL*_ achieves the actual driving time *τ*, i.e., *τ*_*QSL*_ = *τ*, there is no potential capacity for further speedup, or say the speedup evolution can not appear. For intrinsic speedup evolution, it requires *τ*_*QSL*_ < *τ*, and the shorter the *τ*_*QSL*_ the faster the intrinsic speed of evolution (or equivalently, the greater the capacity for potential speedup) will be.

For convenience, we assume that the atom starts in the excited state 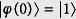, that is 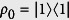. The density matrix of the atom for arbitrary time *t* can be evaluated as^38^ 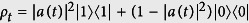. In the light of Eq. (12), the QSL time of the above model can be given by


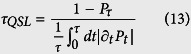


with *P*_*t*_ = |*a(t*)|^2^ denoting the atomic excited state population. In the following we will pay special attention to the problem of the effect of environment coherence and width of the band gap Δ_*c*_ on the QSL time.

**Figure 1:**
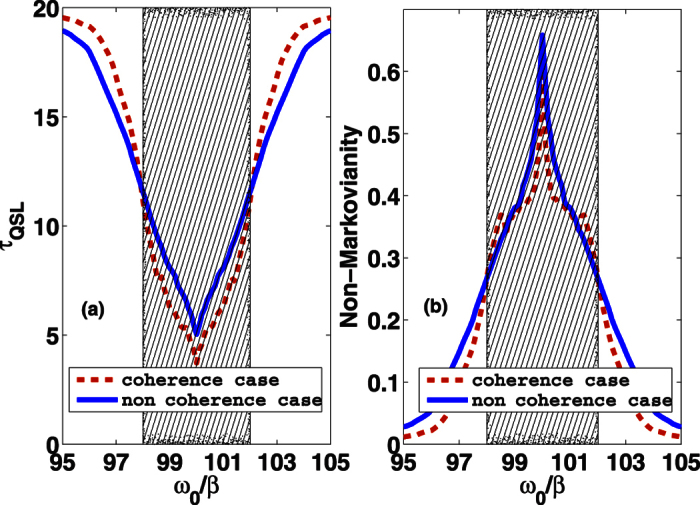
(**a**) The QSL time *τ*_*QSL*_ and (**b**) non-Markovianity 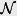 (in unit of 1/*β*) as a function of *ω*_0_/*β*, for the coherence case with *θ*(**r**_0_) = *π*/4 (dashed line) and the non-coherence case (solid line), respectively. Here we set the driving time *τ* = 20 (in unit of 1/*β*). The shadow region refers to the bang gap of the tow-band photonic crystal.

In Fig. 1a, we investigate QSL time *τ*_*QSL*_ versus parameter *ω*_0_. A reasonable comparison between the solutions within and without the environment coherence is clearly shown. Here, we choose the driving time *τ* sufficiently large. This guarantees that the system reaches its steady state. We find that, when *ω*_0_ is in the region of the photonic band gap, *τ*_*QSL*_ of coherence case (dashed line) is smaller than that of the non-coherence case (solid line), which means that the environment coherence can reduce the *τ*_*QSL*_, i.e., increase the intrinsic speed of quantum evolution. While, when *ω*_0_ goes far outside the band gap, the QSL time of coherence increases quickly and becomes larger than that of non-coherence case.

As noted in refs 13 and 10, the non-Markovianity 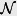 within the driving time can lead to smaller QSL times. In order to further study the relation between the QSL time and the non-Markovianity, we also plot the non-Markovianity for coherence and non-coherence cases. Non-Markovian dynamics, being linked to memory effects of the environment, implies that the lost information flows from environment back to the system^39^. The non-Markovianity 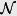 can be defined as the total amount of backflow information, 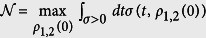, where 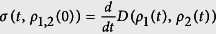 denotes the changing rate of the trace distance. The trace distance is defined as 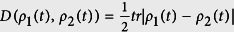 with 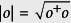. It should be note that the dynamical process is non-Markovian if there exists a pair of initial states such that *σ(t,ρ*_1,2_(0)) > 0, and the maximum is taken over all pairs of initial states. As noted in ref. 40, the pair of optimal states is proved to be the states 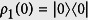 and 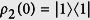. This allows us to derive the rate of change of the trace distance in the form *σ(t,ρ*_1,2_(0)) = ∂_*t*_*P*_*t*_.

The corresponding non-Markovianity 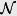 is shown in Fig. 1b. The presence of environmental coherence produces two interesting features. First, except for the regions near the band edges, the non-Markovianity for the non-coherence environment is always greater than the one for the coherence environment. It is implies that environmental coherence can suppress the non-Markovianity. Second, by contrasting Fig. 1a and b, we find that decreasing non-Markovianity will decrease the *τ*_*QSL*_ when *ω*_0_ is near the middle of the band gap, while, decreasing non-Markovianity will increase the QSL time outside the band gap region. Therefore, it is not always true that increasing non-Markovianity can lead to a smaller QSL time.

**Figure 2:**
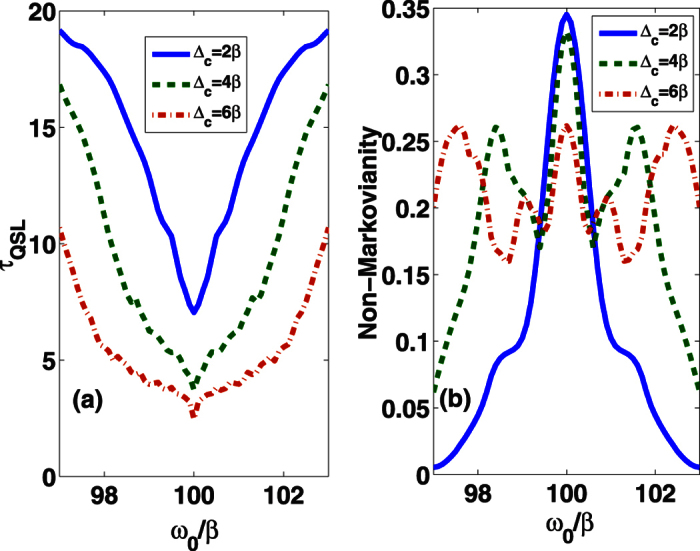
(**a**) The QSL time *τ*_*QSL*_ and (**b**) non-Markovianity 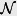 (in unit of 1/*β*) as a function of *ω*_0_/*β* for different values of the width of the band gap Δ_*c*_ with *τ* = 20 (in unit of 1/*β*) and *θ*(**r**_0_) = *π*/4.

Next, we will consider how the QSL time is affected by the width of the band gap Δ_*c*_. Both the *τ*_*QSL*_ and the non-Markovianity for various of Δ_*c*_ are shown in Fig. 2a and b. We observe that the QSL time decreases with the increase of Δ_*c*_. If averaging the non-Markovianity over the presented region of *ω*_0_, one can see that the averaged value of non-Markovianity also decreases with the increasing of Δ_*c*_. Therefore, the reason of the *τ*_*QSL*_ reduction in this case is not due to the enhancement of non-Markovianity.

In concluding this section, we would like to emphasize that, the environmental coherence and the width of the band gap are all play an important role in accelerating the intrinsic speed of the atomic evolution. More importantly, the decrease of the *τ*_*QSL*_ is not directly due to the non-Markovianity, i.e., the total amount of backflow information. What is the mechanism of the intrinsic speedup in a memory environment? What can directly affect the *τ*_*QSL*_ in a reservoir with memory effects? To address the above questions, we first describe the memory effect of the model by using the memory time in the next section.

### Relationship between the QSL time and the memory time

The memory effect of a reservoir connects tightly with the reservoir correlation time, i.e., memory time. It has been shown that the dynamics of an open system is Markovian when the memory time is very short and non-Markovian when the memory time is long^41^. We first describe the memory time of this model.

For a two-level atom with transition frequency near resonant with the band edge of a photonic crystal, a emitted photon will penetrate a localization length^42^ and back. Such a feedback mechanism can result in information backflow, hence non-Markovianity. Also, the time taken by a photon to perform a round trip to the atom should reasonably behave as a memory time, which is a key parameter to the occurrence of memory effect.

Based on this, we take the memory time as *T* = 2*l/v*, where *l* is the localization length and *v* is the photon group velocity. For our two-band PBG reservoir, we will obtain two localization lengths *l*_*l*_ and *l*_*u*_ coming from the lower-band reservoir and the upper-band reservoir. The analytical results of *l*_*u*_ and *l*_*l*_ are shown in the Supplementary Material. The dispersion relation of a two-band PBG environment reads


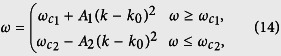


where 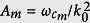 (*m* = 1, 2), and *k*_0_ is a constant characteristic of the dielectric material. Thus, the photon group velocity can be given by *v* = *dω/dk*. To simplify our calculations, we approximate the group velocity as |*v*_*u*_| = |*v*_*l*_| = *χ*, where *χ* is chosen sufficiently small. That is, we assume that the magnitudes of group velocities coming from the upper and lower band reservoirs are the same and equal to *χ*. These approximations are valid because we will focus on processes where the photonic band gap is narrow (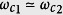) and the atomic frequencies are close to the photonic band edge.

Hence, the memory time is reduced to *T*_*u(l*)_ = 2*l*_*u(l*)_/*χ*, which depends entirely on the localization length *l*_*u(l*)_. If *l*_*u*_ ≥ *l*_*l*_, i.e., *T*_*u*_ ≥ *T*_*l*_, we choose the long time *T*_*u*_ as the memory time of the two-band PBG reservoir, and vice versa. Clearly, the memory time is


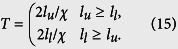


**Figure 3:**
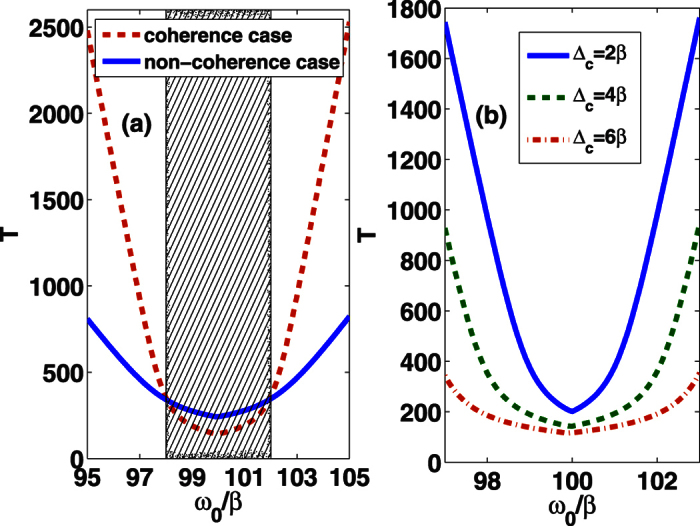
The memory time *T* as a function of *ω*_0_/*β* for (**a**) the coherence case with *θ*(**r**_0_) = *π*/4 (dashed line) and the non-coherence case (solid line), and (**b**) for various values of band-gap width Δ_*c*_. The shadow region refers to the bang gap of the tow-band photonic crystal. Here we set *χ* = 0.1 (in the unit of 1/*β*).

As an illustration, the memory time is plotted as a function of *ω*_0_/*β* in Fig. 3. The comparison between the solutions within and without the environment coherence is shown in Fig. 3a. It is remarkable to find that the memory time exhibits the same behaviors as the QSL time in Fig. 1a. That is, both *τ*_*QSL*_ and *T* of coherence (non-coherence) cases are shorter (longer) than that of non-coherence (coherence) cases for *ω*_0_ is in (out) the band gap region. By contrasting Figs 3b and 2a, the results also confirm that decreasing memory time will decrease the QSL time. We thus conjecture that the memory time could be seen as an essential reflection to the QSL time of the dynamical evolution. The decrease of memory time can make the information return more quickly to the system, which helps to accelerate the intrinsic speed of evolution and therefore lead to a smaller QSL time. On the other hand, from Figs 3b and 2b, we can find that a shorter memory time can cause a smaller non-Markovianity in PBG reservoirs. That is to say, it is not the total amount of backflow information but the backflow rate of information that directly affects the QSL time.

## Discussion

In summary, we have studied a qubit that is coupled to a coherent PBG reservoir. We have investigated how the coherent property and the width of the band gap affect the QSL time of the qubit. We find that the width of the band gap serves to reduce the QSL time. However, the environment coherence can play dual effects. We have also explored the mechanism of the QSL time reduction in our model. It is revealed that the memory time of the reservoir can be seen as an essential reflection to the QSL time.

The ideal physical system to test the phenomenon we illustrated in this work is InAs quantum dots coupled to a planar GaAs photonic crystal^43^. In experiment, to control the embedded position of the quantum dot in order to observe how the QSL time is influenced by the coherent property of the PBG environment, we can use the method of electrohydrodynamic jet printing^44^. For the relevant experimental parameters, the transition frequency and dipole moment of InAs quantum dots are observed to be *ω*_0_ ~ 1.3*PHz* and *d*_0_ ~ 3.3 × 10^−18^*CM*^45^,^46^, respectively, which in turn gives *β* ~ 160 *MHz*. We can tune the *ω*_0_ of quantum dots by the Stark shift with typical shifts Δ ~ 1 *GHz*. Therefore, Δ/*β* ~ 6, and the conditions for intrinsic speedup can be accomplished. Our work may be of theoretical and experimental interests in controlling the QSL time in memory environments.

## Method

### The calculations of Γ_*u*_(*s*) and Γ_*l*_(*s*)

Using the above dispersion relation (Re. (14)), the Laplace transforms of the memory kernels Γ_*u*_(*t* − *τ*) and Γ_*l*_(*t* − *τ*) can be obtained analytically as


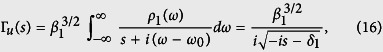



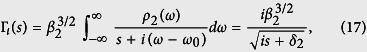


where


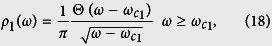



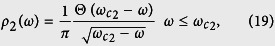


with the Heaviside step function Θ. 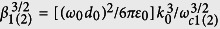 37. *δ*_1(2)_ = *ω*_0_ − *ω*_*c*1(2)_. To keep it simple in this work, we assume 
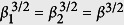
.

## Additional Information

**How to cite this article**: Wang, J. *et al*. Relationship between quantum speed limit time and memory time in a photonic-band-gap environment. *Sci. Rep.* **6**, 39110; doi: 10.1038/srep39110 (2016).

**Publisher's note:** Springer Nature remains neutral with regard to jurisdictional claims in published maps and institutional affiliations.

## Supplementary Material

Supplementary Information

## References

[b1] Anandan J. & Aharonov Y. Geometry of quantum evolution. Phys. Rev. Lett. 65, 1697 (1990). 10042340 10.1103/PhysRevLett.65.1697

[b2] Margolus N. & Levitin L. B. The maximum speed of dynamical evolution. Phys. D 120, 188 (1998).

[b3] Mandelstam L. & Tamm I. The uncertainty relation between energy and time in non-relativistic quantum mechanics. J. Phys. (USSR) 9, 249 (1945).

[b4] del Campo A., Egusquiza I. L., Plenio M. B. & Huelga S. F. Quantum speed limits in open system dynamics. Phys. Rev. Lett. 110, 050403 (2013). 23414008 10.1103/PhysRevLett.110.050403

[b5] Taddei M. M., Escher B. M., Davidovich L. & de Matos Filho R. L. Quantum speed limit for physical processes. Phys. Rev. Lett. 110, 050402 (2013). 23414007 10.1103/PhysRevLett.110.050402

[b6] Marvian I. & Lidar D. A. Quantum speed limits for leakage and decoherence. Phys. Rev. Lett. 115, 210402 (2015). 26636833 10.1103/PhysRevLett.115.210402

[b7] Sun Z., Liu J., Ma J. & Wang X. Quantum speed limits in open systems: Non-Markovian dynamics without rotating-wave approximation. Sci. Rep. 5, 8444 (2015). 25676589 10.1038/srep08444 PMC4649631

[b8] Wu S. X., Zhang Y., Yu C. S. & Song H. S. The initial-state dependence of quantum speed limit. J. Phys. A 48, 045301 (2015).

[b9] Dehdashti S., Bagheri Harouni M., Mirza B. & Chen H. Decoherence speed limit in the spin-deformed boson model. Phys. Rev. A 91, 022116 (2015).

[b10] Zhang Y.-J., Han W., Xia Y.-J., Cao J. P. & Fan H. Quantum speed limit for arbitrary initial states. Sci. Rep. 4, 4890 (2014). 24809395 10.1038/srep04890 PMC4013937

[b11] Xu Z.-Y., Luo S., Yang W.-L., Liu C. & Zhu S. Quantum speedup in a memory environment. Phys. Rev. A 89, 012307 (2014).

[b12] Wei Y.-B., Zou J., Wang Z.-M. & Shao B. Quantum speed limit and a signal of quantum criticality. Sci. Rep. 6, 19308 (2016). 26782296 10.1038/srep19308 PMC4725993

[b13] Deffner S. & Lutz E. Quantum speed limit for non-Markovian dynamics. Phys. Rev. Lett. 111, 010402 (2013). 23862985 10.1103/PhysRevLett.111.010402

[b14] Cimmarusti A. D. . Environment-assisted speed-up of the field evolution in cavity quantum electrodynamics. Phys. Rev. Lett. 114, 233602 (2015). 26196802 10.1103/PhysRevLett.114.233602

[b15] Meng X., Wu C. & Guo H. Minimal evolution time and quantum speed limit of non-Markovian open systems. Sci. Rep. 5, 16357 (2015). 26565062 10.1038/srep16357 PMC4643350

[b16] Liu C., Xu Z. Y. & Zhu S. Quantum-speed-limit time for multiqubit open systems. Phys. Rev. A 91, 022102 (2015).

[b17] Zhang Y.-J. . Classical-driving-assisted quantum speed-up. Phys. Rev. A 91, 032112 (2015).

[b18] Liu H. B., Yang W. L., An J. H. & Xu Z. Y. Mechanism for quantum speedup in open quantum systems. Phys. Rev. A 93, 020105 (2016).

[b19] John S. Electromagnetic absorption in a disordered medium near a photon mobility edge. Phys. Rev. Lett. 53, 2169 (1984).

[b20] John S. & Wang J. Quantum electrodynamics near a photonic band gap: photo bound states and dressed atoms. Phys. Rev. Lett. 64 2418(1990). 10041707 10.1103/PhysRevLett.64.2418

[b21] John S. & Wang J. Quantum optics of localized light in a photonic band gap. Phys. Rev. B 43 12772 (1991). 10.1103/physrevb.43.12772 9997091

[b22] de Vega I., Alonso D. & Gaspard P. Two-level system immersed in a photonic band-gap material: A non-Markovian stochastic Schrödinger-equation approach. Phys. Rev. A 71, 023812 (2005).

[b23] Bay S., Lambropoulos P. & Molmer K. Atom-atom interaction in strongly modified reservoirs. Phys. Rev. A 55, 1485 (1997).

[b24] Lecamp G., Lalanne P. & Hugonin J. P. Very Large Spontaneous-Emission *β* Factors in Photonic-Crystal Waveguides. Phys. Rev. Lett. 99, 023902 (2007). 17678224 10.1103/PhysRevLett.99.023902

[b25] Wu J.-N., Huang C.-H., Cheng S.-C. & Hsieh W.-F. Spontaneous emission from a two-level atom in anisotropic one-band photonic crystals: A fractional calculus approach. Phys. Rev. A 81, 023827 (2010).

[b26] Bellomo B., Lo Franco R., Maniscalco S. & Compagno G. Entanglement trapping in structured environments. Phys. Rev. A 78, 060302(R) (2008).

[b27] Wu Y. N., Wang J. & Zhang H. Z. Threshold for formation of atom-photon bound states in a coherent photonic band-gap reservoir. Opt. Commun. 366, 431 (2016).

[b28] Bellomo B., Lo Franco R., Maniscalco S. & Compagno G. Two-qubit entanglement dynamics for two different non-Markovian environments. Phys. Scr. T 140, 014014 (2010).

[b29] Bellomo B., Lo Franco R. & Compagno G. Long-time protection of nonlocal entanglement. Adv. Sci. Lett. 2, 459 (2009).

[b30] Lo Franco R., Bellomo B., Maniscalco S. & Compagno G. Dynamics of quantum correlations in two-qubit systems within non-Markovian environments. Int. J. Mod. Phys. B 27, 1345053 (2013).

[b31] Man Z.-X., Xia Y.-J. & Lo Franco R. Cavity-based architecture to preserve quantum coherence and entanglement. Sci. Rep. 5, 13843 (2015). 26351004 10.1038/srep13843 PMC4563358

[b32] Zhang Y.-J., Han W., Fan H. & Xia Y.-J. Enhancing entanglement trapping by weak measurement and quantum measurement reversal. Ann. Phys. 354, 203 (2015).

[b33] Li H., Xie S., Xu J. & Yang Y. Effects of engineering initial states and quantum interference near the edge of a photonic bandgap on the entanglement. J. Opt. Soc. Am. B 32, 1050 (2015).

[b34] Aolita L., de Melo F. & Davidovich L. Open-system dynamics of entanglement: a key issues review. Rep. Prog. Phys. 78, 042001 (2015). 25811809 10.1088/0034-4885/78/4/042001

[b35] Tufarelli T., Kim M. S. & Ciccarello F. Non-Markovianity of a quantum emitter in front of a mirror. Phys. Rev. A 90, 012113 (2014).

[b36] Cheng S.-C., Wu J.-N., Yang T.-J. & Hsieh W.-F. Effect of atomic position on the spontaneous emission of a three-level atom in a coherent photonic-band-gap reservoir. Phys. Rev. A 79, 013801 (2009).

[b37] Yang Y., Fleischhauer M. & Zhu S.-Y. Spontaneous emission from a two-level atom in two-band anisotropic photonic crystals. Phys. Rev. A 68, 043805 (2003). 10.1103/PhysRevE.68.015602 12935192

[b38] Breuer H.-P. & Petruccione F. The Theory of Open Quantum Systems (Oxford University Press, Oxford, UK, 2002).

[b39] Breuer H.-P., Laine E.-M. & Piilo J. Measure for the degree of non-Markovian behavior of quantum processes in open systems. Phys. Rev. Lett. 103, 210401 (2009). 20366019 10.1103/PhysRevLett.103.210401

[b40] Wißmann S., Karlsson A., Laine E.-M., Piilo J. & Breuer H.-P. Optimal state pairs for non-Markovian quantum dynamics. Phys. Rev. A 86, 062108 (2012).

[b41] Laine E.-M., Piilo J. & Breuer H.-P. Measure for the non-Markovianity of quantum processes. Phys. Rev. A 81, 062115 (2010).

[b42] John S. & Quang T. Photon-hopping conduction and collectively induced transparency in a photonic band gap. Phys. Rev. A 52, 4083 (1995). 9912723 10.1103/physreva.52.4083

[b43] Yoshie T. . Vacuum Rabi splitting with a single quantum dot in a photonic crystal nanocavity. Nature 432, 200 (2004). 15538363 10.1038/nature03119

[b44] See G.-G. . Polarized quantum dot emission in electrohydrodynamic jet printed photonic crystal. Appl. Rhys. Lett. 107, 051101 (2015).

[b45] Alėn B., Bickel F., Karraib K., Warburton R.-J. & Petroff P.-M. Stark-shift modulation absorption spectroscopy of single quantum dots. Appl. Phys. Lett. 83, 2235 (2003).

[b46] Yang Y. & Zhu S.-Y. Spontaneous-emission enhancement and population oscillation in photonic crystals via quantum interference. Phys. Rev. A 61, 043809 (2000).

