# Supplementary material for: Relationship between quantum speed limit time and memory time in a photonic-band-gap environment
Source: Sci Rep. 2016 Dec 23;6:39110. doi: 10.1038/srep39110 (PMC5180220; doi:10.1038/srep39110)
Supplement: Supplementary Information [file srep39110-s1.pdf]

Relationship between quantum speed limit time and memory time in a photonic-band-gap environment

J. Wang[1†], Y. N. Wu[2], M. L. Mo[1], H. Z. Zhang[2]

[1] *School of physics and technology, University of Jinan, Jinan, 250022, China*

[2] *College of physics, Jilin University, Changchun, 130023, China*

[†] *To whom correspondence should be addressed. E-mail: sps_wangj@ujn.edu.cn*

Supplementary information

The calculations of probability amplitudes. In this section we calculate the amplitudes $a\left(t\right)$ and $a_{no}\left(t\right)$ for coherence and non-coherence cases, respectively. For convenience, several functions are defined as follows:

$$f_1\left(x\right)=1-\sin^2\theta\left(\mathbf{r}_0\right)\cos^2\theta\left(\mathbf{r}_0\right)\begin{bmatrix} G^{-1}\left(x+i\Delta_c\right)\frac{i\beta^{3/2}}{\sqrt{ix+\delta_1}} \\ +G^{-1}\left(x-i\Delta_c\right)\frac{\beta^{3/2}}{\sqrt{-ix-\delta_2}}\end{bmatrix},$$

$$f_2\left(x\right)=1-\sin^2\theta\left(\mathbf{r}_0\right)\cos^2\theta\left(\mathbf{r}_0\right)\begin{bmatrix} H^{-1}\left(x+i\Delta_c\right)\frac{\beta^{3/2}}{\sqrt{-ix-\delta_1}} \\ +H^{-1}\left(x-i\Delta_c\right)\frac{\beta^{3/2}}{\sqrt{-ix-\delta_2}}\end{bmatrix},$$

$$f_3\left(x\right)=1-\sin^2\theta\left(\mathbf{r}_0\right)\cos^2\theta\left(\mathbf{r}_0\right)\begin{bmatrix} K^{-1}\left(x+i\Delta_c\right)\frac{i\beta^{3/2}}{\sqrt{ix+\delta_1}} \\ +K^{-1}\left(x-i\Delta_c\right)\frac{\beta^{3/2}}{-i\sqrt{ix+\delta_2}}\end{bmatrix},$$

$$G(x)=x+\frac{\beta^{3/2}}{i\sqrt{-ix-\delta_1}}\cos^4\theta\left(\mathbf{r}_0\right)+\frac{i\beta^{3/2}}{\sqrt{ix+\delta_2}}\sin^4\theta\left(\mathbf{r}_0\right),$$

$$G_{no}(x)=x+\frac{\beta^{3/2}}{i\sqrt{-ix-\delta_1}}+\frac{i\beta^{3/2}}{\sqrt{ix+\delta_2}},$$

$$H(x) = x + \frac{\beta^{3/2}}{i\sqrt{-ix-\delta_1}}\cos^4\theta(\mathbf{r}_0) + \frac{\beta^{3/2}}{\sqrt{-ix-\delta_2}}\sin^4\theta(\mathbf{r}_0),$$

$$H_{no}(x) = x + \frac{\beta^{3/2}}{i\sqrt{-ix-\delta_1}} + \frac{\beta^{3/2}}{\sqrt{-ix-\delta_2}},$$

$$K(x) = x + \frac{\beta^{3/2}}{\sqrt{ix+\delta_1}}\cos^4\theta(\mathbf{r}_0) + \frac{i\beta^{3/2}}{\sqrt{ix+\delta_2}}\sin^4\theta(\mathbf{r}_0),$$

$$K_{no}(x) = x + \frac{\beta^{3/2}}{\sqrt{ix+\delta_1}} + \frac{i\beta^{3/2}}{\sqrt{ix+\delta_2}},$$

$$N(x) = -x + i\delta_2 + \frac{\beta^{2/3}}{\sqrt{ix+\Delta_c}}\cos^4\theta(\mathbf{r}_0),$$

$$N_{no}(x) = -x + i\delta_2 + \frac{\beta^{2/3}}{\sqrt{ix+\Delta_c}},$$

$$M(x) = -x + i\delta_1 + \frac{i\beta^{3/2}}{\sqrt{-ix+\Delta_c}}\sin^4\theta(\mathbf{r}_0),$$

$$M_{no}(x) = -x + i\delta_1 + \frac{i\beta^{3/2}}{\sqrt{-ix+\Delta_c}},$$

$$\eta_1(x) = \frac{f_3(-x+i\delta_1)}{M(x) + \frac{\beta^{2/3}}{-i\sqrt{-ix}}\cos^4\theta(\mathbf{r}_0)} - \frac{f_1(-x+i\delta_1)}{M(x) + \frac{\beta^{2/3}}{\sqrt{ix}}\cos^4\theta(\mathbf{r}_0)},$$

$$\eta_2(x) = \frac{f_1(-x+i\delta_2)}{N(x) + \frac{i\beta^{2/3}}{\sqrt{-ix}}\sin^4\theta(\mathbf{r}_0)} - \frac{f_2(-x+i\delta_2)}{N(x) + \frac{\beta^{2/3}}{\sqrt{ix}}\sin^4\theta(\mathbf{r}_0)}.$$

Through performing the inverse Laplace transform, the amplitude $a(t)$ of coherence case can be written as

$$a(t) = \frac{1}{2\pi i}\int_{\sigma-i\infty}^{\sigma+i\infty}\tilde{a}(s)\,e^{st}ds, \tag{S1}$$

where $\tilde{a}(s)$ is given by Eq. (12). σ is the real number which satisfies demand that $s=\sigma$ lies to the right of all the singular points of $\tilde{a}(s)$. It is readily checked that $s=i\delta_{1(2)}$ are branch points of $\tilde{a}(s)$. Using the residue theorem and the integration contour shown in Fig. (S1), we have

$$a(t) = \sum_j \frac{f_1\left(x_j^{(1)}\right)}{G'\left(x_j^{(1)}\right)}e^{x_j^{(1)}t} - \frac{1}{2\pi i}\left[\int_{c_3}+\int_{c_4}+\int_{c_6}+\int_{c_7}\right]\frac{f_1(x)}{G(x)}e^{xt}dx. \tag{S2}$$

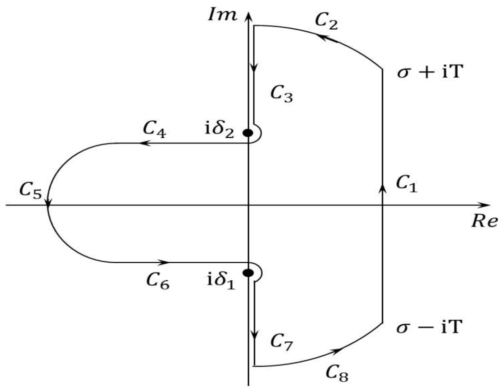

Fig. S 1: The integral curve used in the equation (S2).

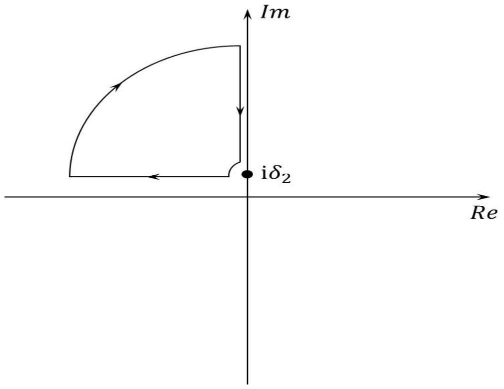

Fig. S 2: The integral curve used in the equation (S3).

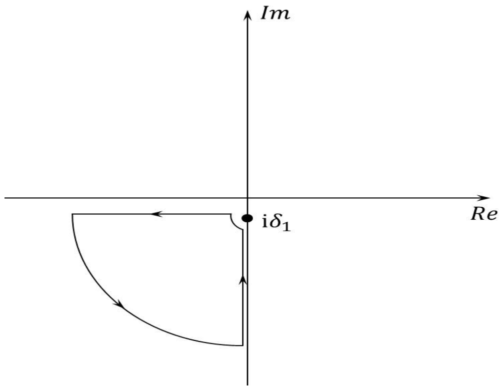

Fig. S 3: The integral curve used in the equation (S6).

Here $x_j^{(1)}$ represent the solutions of $G(x) = 0$ in the region $[Re\,(x) > 0]$ or $[\delta_1 < Im\,(x) < \delta_2]$, $G'\,(x)$ is the derivative of the function $G\,(x)$.

Using the Fig. (S2), the integral along c_3 yields

$$\frac{1}{2\pi i}\int_{c3}\frac{f_1\,(x)}{G\,(x)}e^{xt}dx = \frac{1}{2\pi i}\int_{i\infty}^{i\delta_2}\frac{f_2\,(x)}{H\,(x)}e^{xt}dx = -\sum_j \frac{f_2\left(x_j^{(2)}\right)}{H'\left(x_j^{(2)}\right)}e^{x_j^{(2)}t} \tag{S3}$$

$$-\frac{e^{i\delta_2 t}}{2\pi i}\int_0^{-\infty}\frac{1-\sin^2\theta\,(\mathbf{r}_0)\cos^2\theta\,(\mathbf{r}_0)\begin{bmatrix} H^{-1}\,(x+i\,(\Delta_c+\delta_2))\,\frac{i\beta^{2/3}}{i\sqrt{-ix+\Delta_c}} \\ +H^{-1}\,(x+i\delta_1)\,\frac{\beta^{2/3}}{i\sqrt{-ix}}\end{bmatrix}}{x+i\delta_2+\frac{\beta^{2/3}}{\sqrt{-ix+\Delta_c}}\cos^4\theta\,(\mathbf{r}_0)+\frac{\beta^{2/3}}{\sqrt{-ix}}\sin^4\theta\,(\mathbf{r}_0)}e^{xt}dx,$$

where $x_j^{(2)}$ represent the solutions of $H(x) = 0$ in the region $[Re\,(x) < 0\ and\ Im\,(x) > \delta_2]$.

The integral along c_4 is

$$\frac{1}{2\pi i}\int_{c4}\frac{f_1\,(x)}{G\,(x)}e^{xt}dx = \frac{e^{i\delta_2 t}}{2\pi i}\int_0^{-\infty}\frac{f_1\,(x+i\delta_2)}{G\,(x+i\delta_2)}e^{xt}dx \tag{S4}$$

$$= \frac{e^{i\delta_2 t}}{2\pi i}\int_0^{-\infty}\frac{1-\sin^2\theta\,(\mathbf{r}_0)\cos^2\theta\,(\mathbf{r}_0)\begin{bmatrix} G^{-1}\,(x+i\,(\Delta_c+\delta_2))\,\frac{i\beta^{2/3}}{\sqrt{ix-\Delta_c}} \\ +G^{-1}\,(x+i\delta_1)\,\frac{\beta^{2/3}}{\sqrt{-ix}}\end{bmatrix}}{x+i\delta_2+\frac{\beta^{2/3}}{\sqrt{-ix+\Delta_c}}\cos^4\theta\,(\mathbf{r}_0)+\frac{i\beta^{2/3}}{\sqrt{ix}}\sin^4\theta\,(\mathbf{r}_0)}e^{xt}dx.$$

The integral along c_6 is

$$\frac{1}{2\pi i}\int_{c_6}\frac{f_1(x)}{G(x)}e^{xt}dx=\frac{e^{i\delta_1 t}}{2\pi i}\int_{-\infty}^{0}\frac{f_1(x+i\delta_1)}{G(x+i\delta_1)}e^{xt}dx \tag{S5}$$

$$=\frac{e^{i\delta_1 t}}{2\pi i}\int_{-\infty}^{0}\frac{1-\sin^2\theta(\mathbf{r}_0)\cos^2\theta(\mathbf{r}_0)\left[\begin{array}{c}G^{-1}(x+i\delta_2)\frac{\beta^{3/2}}{\sqrt{-ix}}+\\ G^{-1}(x+i(\delta_1-\Delta_c))\frac{\beta^{2/3}}{\sqrt{-ix-\Delta_c}}\end{array}\right]}{x+i\delta_1+\frac{\beta^{2/3}}{\sqrt{-ix}}\cos^4\theta(\mathbf{r}_0)+\frac{i\beta^{3/2}}{\sqrt{ix+\Delta_c}}\sin^4\theta(\mathbf{r}_0)}e^{xt}dx.$$

Using the Fig. (S3), the integral along c_7 yields

$$\frac{1}{2\pi i}\int_{c_7}\frac{f_1(x)}{G(x)}e^{xt}dx=\frac{1}{2\pi i}\int_{i\delta_1}^{-\infty i}\frac{f_3(x)}{K(x)}e^{xt}dx=-\sum_j\frac{f_3\left(x_j^{(3)}\right)}{K'\left(x_j^{(3)}\right)}e^{x_j^{(3)}t} \tag{S6}$$

$$-\frac{e^{i\delta_1 t}}{2\pi i}\int_{-\infty}^{0}\frac{1-\sin^2\theta(\mathbf{r}_0)\cos^2\theta(\mathbf{r}_0)\left[\begin{array}{c}K^{-1}(x+i\delta_2)\frac{i\beta^{3/2}}{\sqrt{ix}}+\\ K^{-1}(x+i(\delta_1-\Delta_c))\frac{\beta^{2/3}}{-i\sqrt{ix+\Delta_c}}\end{array}\right]}{x+i\delta_1+\frac{\beta^{2/3}}{-i\sqrt{ix}}\cos^4\theta(\mathbf{r}_0)+\frac{i\beta^{3/2}}{\sqrt{ix+\Delta_c}}\sin^4\theta(\mathbf{r}_0)}e^{xt}dx.$$

In Eq. (S6), $x_j^{(3)}$ represent the solutions of $K(x)=0$ in the region $[Re\,(x)<0\ and\ Im\,(x)<\delta_1]$.

By substituting Eqs. (S3–S6) into Eq. (S2), we then obtain

$$\begin{aligned}a(t)&=\sum_j\frac{f_1\left(x_j^{(1)}\right)}{G'\left(x_j^{(1)}\right)}e^{x_j^{(1)}t}+\sum_j\frac{f_2\left(x_j^{(2)}\right)}{H'\left(x_j^{(2)}\right)}e^{x_j^{(2)}t}+\sum_j\frac{f_3\left(x_j^{(3)}\right)}{K'\left(x_j^{(3)}\right)}e^{x_j^{(3)}t}\\&+\frac{1}{2\pi i}\int_0^{\infty}\left[\eta_1(x)e^{i\delta_1 t}+\eta_2(x)e^{i\delta_2 t}\right]e^{-xt}dx.\end{aligned} \tag{S7}$$

For non-coherence case, the Laplace transform of $a_{no}(t)$ can be given by [1]

$$\tilde{a}_{no}(s)=\left[s+\Gamma_u(s)+\Gamma_\iota(s)\right]^{-1}. \tag{S8}$$

With the help of the residue theorem and complex function integration, we can obtain the expression of $a_{no}(t)$ as

$$a_{no}\left(t\right)=\sum_{m}\frac{e^{x_m^{(1)}t}}{G'_{no}\left(x_m^{(1)}\right)}+\sum_{m}\frac{e^{x_m^{(2)}t}}{H'_{no}\left(x_m^{(2)}\right)}+\sum_{m}\frac{e^{x_m^{(3)}t}}{K'_{no}\left(x_m^{(3)}\right)}$$
$$+\frac{e^{i\delta_1 t}}{2\pi i}\int_0^\infty\left[\frac{1}{M_{no}\left(x\right)+\frac{\beta^{2/3}}{-i\sqrt{-ix}}}-\frac{1}{M_{no}\left(x\right)+\frac{\beta^{2/3}}{\sqrt{ix}}}\right]e^{-xt}dx$$
$$+\frac{e^{i\delta_2 t}}{2\pi i}\int_0^\infty\left[\frac{1}{N_{no}\left(x\right)+\frac{i\beta^{2/3}}{\sqrt{-ix}}}-\frac{1}{N_{no}\left(x\right)+\frac{\beta^{2/3}}{\sqrt{ix}}}\right]e^{-xt}dx, \quad \text{(S9)}$$

where $x_m^{(1)}$ represent the solutions of $G_{no}(x)=0$ in the region $[Re\left(x\right)>0]$ or $[\delta_1<Im\left(x\right)<\delta_2]$, $x_m^{(2)}$ represent the solutions of $H_{no}(x)=0$ in the region $[Re\left(x\right)<0\ and\ Im\left(x\right)>\delta_2]$ and $x_m^{(3)}$ are the solutions of $K_{no}(x)=0$ in the region $[Re\left(x\right)<0\ and\ Im\left(x\right)<\delta_1]$.

The calculations of the localization lengths l_u and l_l. From the expression of $a\left(t\right)$ given by Eq. (S7), we can find that the number and characteristics of roots $x_j^{(1)}$, $x_j^{(2)}$ and $x_j^{(3)}$ are important in the population dynamics and the emitted field. When the purely imaginary roots for $G(x)=0$ exist, population is trapped in the atomic upper level as $t\rightarrow\infty$, and the emitted field contains localized mode(s), which does not decay in time and has localization lengths. In what follows, we calculate the localized field emitted by the atom.

The amplitude of the radiation field coming from the contribution of the upper (lower) band at a space point $\mathbf{r}$ can be given by [2]

$$F_{u(l)}(\mathbf{r},\mathbf{t})=\frac{\omega_0 d_0\sin\eta}{8\pi^2\varepsilon_0 ri}\int\limits_0^\infty\frac{c_{u(l)}\left(\mathbf{r}_0,t\right)}{g_{u(l)}\left(\mathbf{r}_0\right)}e^{-i(\omega_{u(l)}t-kr)}dk, \quad \text{(S10)}$$

where η is the angle between the $\mathbf{r}$ vector and the atomic dipole vector. The above equation is valid in the far field and the radiation field is polarized in the x direction. The amplitude $c_{u(l)}\left(\mathbf{r}_0,t\right)$ can be calculated from $a\left(t\right)$. Due to the four terms of $a\left(t\right)$ given by Eq. (S7), the radiation field can also be written as the sum of four parts,

$$F_{u(l)}(\mathbf{r},\mathbf{t})=F_{u(l)}^{(1)}(\mathbf{r},\mathbf{t})+F_{u(l)}^{(2)}(\mathbf{r},\mathbf{t})+F_{u(l)}^{(3)}(\mathbf{r},\mathbf{t})+F_{u(l)}^{(4)}(\mathbf{r},\mathbf{t}), \quad \text{(S11)}$$

where the $F_{u(l)}^{(1)}(\mathbf{r},\mathbf{t})$ stems from the pure imaginary roots $x_j^{(1)}$, $F_{u(l)}^{(2)}(\mathbf{r},\mathbf{t})$ and $F_{u(l)}^{(3)}(\mathbf{r},\mathbf{t})$ come from the complex roots $x_j^{(2)}$ and $x_j^{(3)}$, $F_{u(l)}^{(4)}(\mathbf{r},\mathbf{t})$ comes from the last integral terms of $a\,(t)$ and always exists. If we have only one pure imaginary root $x_1^{(1)} = iy_1$ (y is a real number), $F_{u(l)}^{(2)}(\mathbf{r},\mathbf{t})$ and $F_{u(l)}^{(3)}(\mathbf{r},\mathbf{t})$ will be replaced by zero, and $F_{u(l)}(\mathbf{r},\mathbf{t}) = F_{u(l)}^{(1)}(\mathbf{r},\mathbf{t}) + F_{u(l)}^{(4)}(\mathbf{r},\mathbf{t})$. Here, we only discuss the case of having only one pure imaginary root.

The term of $a\,(t)$ related to the pure imaginary root $x_j^{(1)}$ is $\frac{f_1\left(x_j^{(1)}\right)}{G'\left(x_j^{(1)}\right)} e^{x_j^{(1)}t}$. From the Eq. (S10), the field $F_u^{(1)}(\mathbf{r},\mathbf{t})$ coming from the contribution of the upper band can be obtained as

$$\begin{aligned} F_u^{(1)}(\mathbf{r},\mathbf{t}) &= -\frac{\omega_0 d_0 \sin\eta}{8\pi\varepsilon_0 r}\frac{k_0^2}{\omega_{c_1}}\frac{f_1\left(x_j^{(1)}\right)}{G'\left(x_j^{(1)}\right)} \times (\sqrt{\frac{\omega_{c_1}}{\omega_{c_1}-(\omega_0-y_1)}}+i)e^{-i(\omega_0-y_1)t+ik_0 r} \\ &\quad \times\Theta(\sqrt{\frac{\omega_{c_1}-(\omega_0-y_1)}{\omega_{c_1}}}t-\frac{k_0 r}{2\omega_{c_1}}) \times \exp(-k_0 r\sqrt{\frac{\omega_{c_1}-(\omega_0-y_1)}{\omega_{c_1}}}). \end{aligned} \tag{S12}$$

Similarly, the emitted field $F_l^{(1)}(\mathbf{r},\mathbf{t})$ from the contribution of the lower band is

$$\begin{aligned} F_l^{(1)}(\mathbf{r},\mathbf{t}) &= -\frac{\omega_0 d_0 \sin\eta}{8\pi\varepsilon_0 r}\frac{k_0^2}{\omega_{c_2}}\frac{f_1\left(x_j^{(1)}\right)}{G'\left(x_j^{(1)}\right)} \times (\sqrt{\frac{\omega_{c_2}}{(\omega_0-y_1)-\omega_{c_2}}}+i)e^{-i(\omega_0-y_1)t+ik_0 r} \\ &\quad \times\Theta(\sqrt{\frac{(\omega_0-y_1)-\omega_{c_2}}{\omega_{c_2}}}t-\frac{k_0 r}{2\omega_{c_2}}) \times \exp(-k_0 r\sqrt{\frac{(\omega_0-y_1)-\omega_{c_2}}{\omega_{c_2}}}). \end{aligned} \tag{S13}$$

It is clear that the amplitudes of the fields $F_u^{(1)}(\mathbf{r},\mathbf{t})$ and $F_l^{(1)}(\mathbf{r},\mathbf{t})$ do not decay in time. The fields $F_u^{(1)}(\mathbf{r},\mathbf{t})$ and $F_l^{(1)}(\mathbf{r},\mathbf{t})$ represent localized fields, and drop exponentially with increasing distance from the atom as e^{-r/l_u} and e^{-r/l_l}, respectively. Here $l_u = \sqrt{\frac{\omega_{c_1}}{\omega_{c_1}-(\omega_0-y_1)}}$ and $l_l = \sqrt{\frac{\omega_{c_2}}{(\omega_0-y_1)-\omega_{c_2}}}$ are the localization lengths coming from the contribution of the upper and lower band reservoirs. If two pure imaginary roots iy_1 and iy_2 exist, with $y_1 > y_2$, the localization lengths satisfy $l_{u_1} < l_{u_2}$ and $l_{l_1} > l_{l_2}$.

1. Yang, Y., Fleischhauer, M. & Zhu, S. Y. Spontaneous emission from a two-level atom in two-band anisotropic photonic crystals. *Phys. Rev. A* **68**, 043805 (2003).

2. Yang, Y. & Zhu, S. Y. Spontaneous-emission enhancement and population oscillation in photonic crystals via quantum interference. *Phys. Rev. A* **61**, 043809 (2000).
